# Prior Evidence of Putative Novel *Rhinovirus* Species, Australia

**DOI:** 10.3201/eid1411.080725

**Published:** 2008-11

**Authors:** Ian M. Mackay, Stephen B. Lambert, Peter K. McErlean, Cassandra E. Faux, Katherine E. Arden, Michael D. Nissen, Theo P. Sloots

**Affiliations:** Sir Albert Sakzewski Virus Research Centre, Herston, Queensland, Australia; University of Queensland, Brisbane, Queensland, Australia

**Keywords:** human rhinovirus C, genotype, respiratory tract infection, Australia, letter

## To the Editor

 Briese et al. (*1*) are to be congratulated for their delineation of the global geographic presence of human rhinovirus (HRV) strains similar to those reported in 2006 from one third of cases of an otherwise pathogen-negative respiratory outbreak in New York. Of equal importance is the temporal occurrence of these strains. Although it is intriguing to suggest, on the basis of limited sequence data, that these strains were circulating at least 2 centuries earlier (*1*), Briese et al. neglect to acknowledge empirical evidence that what we now call HRV-C strains circulated before 2004–2005. Unculturable PCR-positive rhinoviruses were reported in 1993; however, more compelling is the fact that subgenomic sequence and phylogenetic data were reported from Belgium (*2*), Australia (*3*), and then New York (*4*). The Belgium noncoding sequences were reported in 2006 but originated from specimens collected in 1998–1999. Australian coding sequences from 2003 to 2004 were assigned, for the first time, to a novel clade called HRV-A2, reflecting both their phylogenetic isolation and branching from the known HRV-A strains (*3*).

It can be deduced that NY-041 and NY-060, strains from the 2004 New York winter outbreak, are variants (>98% amino acid identity) of the first characterized HRV-A2 strain, HRV-QPM (*4*,*5*). More recently, we proposed that the HRV-A2 strains diverged sufficiently to meet several of the International Committee on Taxonomy of Viruses criteria for classifying a putative new species, HRV-C (*6*).

It is an exciting time for those interested in rhinoviruses. With increased implementation of multiplexed screening approaches (such as the MassTag PCR), or by simply including a specific and sensitive PCR for all known strains (*7*), further details of the geographic and temporal extent of the neglected rhinoviruses should soon be available. Better identification may finally enable accurate characterization of the clinical, economic, and social impact (*8*) of HRV infection.
