# Supplementary material for: Prior Evidence of Putative Novel Rhinovirus Species, Australia
Source: Emerg Infect Dis. 2008 Nov;14(11):1823–5. doi: 10.3201/eid1411.080725 (PMC2630754; doi:10.3201/eid1411.080725)
Supplement: Related texts

# Reply: In Response

**Authors:** Thomas Briese, Gustavo Palacios, W. Ian Lipkin, Neil Renwick, Marietjie Venter, Richard G. Jarman, Samuel R. Dominguez, Kathryn V. Holmes, Edward C. Holmes

**Affiliations:** Columbia University, New York, New York, USA (T. Briese, G. Palacios, W.I. Lipkin); Rockefeller University, New York (N. Renwick); University of Pretoria, Pretoria, South Africa *(*M. Venter), Armed Forces Research Institute of Medical Sciences, Bangkok, Thailand (R.G. Jarman); University of Colorado Denver School of Medicine, Aurora, Colorado, USA (S.R. Dominguez, K.V. Holmes); Pennsylvania State University, University Park, Pennsylvania, USA (E.C. Holmes); Fogarty International Center, Bethesda, Maryland, USA (E.C. Holmes)

We appreciate the enthusiasm for our recent publication highlighting the global distribution of a long-unrecognized third clade of rhinoviruses. Robust, sequence-based clock estimates with associated confidence limits indicate that these viruses have been circulating for hundreds of years (*1*), consistent with the presence of such viruses in historic samples. As isolates from various collections are analyzed in informative regions (e.g., virus protein [VP] 4/2 or VP1), we will undoubtedly find examples in which human rhinoviruses (HRVs) could have been classified as members of the new species HRV-C but were not because the characteristics that define HRV-C were not yet appreciated or because only noncoding sequences had been analyzed. Indeed, we anticipate that waxing interest in HRVs may well lead to the discovery of additional clades.

There has been discussion in the field as to whether the novel sequences represent a sublineage HRV-A2 of the classified species HRV-A (*2*,*3*), as Mackay et al. had proposed, or whether they should be considered as representatives of a third species of HRV (*4*,*5*). The International Committee on Taxonomy of Viruses (ICTV) is charged with the recognition and naming of taxonomic entities. Thus, we provisionally designated our sequences as a novel clade distinct from HRV-A and HRV-B (*4*) and submitted a proposal to ICTV with data supporting the recognition of HRV-C as a third species of rhinovirus. The proposal was recently approved by the ICTV Study Group on Picornaviruses (Europic May 2008 meeting in Sitges, Spain). Irrespective of taxonomic discourse, we agree with Mackay and colleagues that molecular analyses of as-yet-uncultured HRVs are fascinating and have potential to reveal unexpected insights into the role of HRVs in disease.

## References

[1] Briese T, Renwick N, Venter M, Jarman RG, Ghosh D, Kondgen S, Global distribution of novel rhinovirus genotype. Emerg Infect Dis. 2008;14:944–7.1850791010.3201/eid1406.080271PMC2600308

[2] McErlean P, Shackelton LA, Lambert SB, Nissen MD, Sloots TP, Mackay IM. Characterisation of a newly identified human rhinovirus, HRV-QPM, discovered in infants with bronchiolitis. J Clin Virol. 2007;39:67–75. 10.1016/j.jcv.2007.03.01217482871PMC7172271

[3] Lee WM, Kiesner C, Pappas T, Lee I, Grindle K, Jartti T, A diverse group of previously unrecognized human rhinoviruses are common causes of respiratory illnesses in infants. PLoS One. 2007;2:e966. 10.1371/journal.pone.000096617912345PMC1989136

[4] Lamson D, Renwick N, Kapoor V, Liu Z, Palacios G, Ju J, MassTag polymerase-chain-reaction detection of respiratory pathogens, including a new rhinovirus genotype, that caused influenza-like illness in New York State during 2004–2005. J Infect Dis. 2006;194:1398–402. 10.1086/50855117054069PMC7110122

[5] Lau SK, Yip CC, Tsoi HW, Lee RA, So LY, Lau YL, Clinical features and complete genome characterization of a distinct human rhinovirus (HRV) genetic cluster, probably representing a previously undetected HRV species, HRV-C, associated with acute respiratory illness in children. J Clin Microbiol. 2007;45:3655–64. 10.1128/JCM.01254-0717804649PMC2168475
